# Is periodontitis associated with obstructive sleep apnea? A systematic review and meta-analysis

**DOI:** 10.4317/jced.59478

**Published:** 2022-04-01

**Authors:** Nazanin Khodadadi, Mehrnaz Khodadadi, Mohammad Zamani

**Affiliations:** 1 Student Research Committee, Faculty of Dentistry, Babol University of Medical Sciences, Babol, Iran; 2 Ayatollah Amoli Branch, Islamic Azad University, Amol, Iran; 3 Student Research Committee, Babol University of Medical Sciences, Babol, Iran

## Abstract

**Background:**

There are conflicting results on the association between periodontitis and obstructive sleep apnea (OSA). In the present study, we performed a contemporaneous systematic review and meta-analysis to clarify this issue. We also tried to find out whether periodontitis is associated with OSA severity.

**Material and Methods:**

We searched the literature published from the inception to 31 December 2021 from the databases of Embase, PubMed, Scopus, and Web of Science, using a combination of suitable keywords, without language restriction. We included observational studies recruiting adults (≥18 years old) that evaluated the association between periodontitis and OSA. Two investigators screened independently the titles and abstracts of the identified articles for potential suitability. We compared the prevalence of periodontitis between OSA-positive and negative individuals using a pooled odds ratio (OR) with a 95% confidence interval (CI). The heterogeneity between the studies was examined by the I2 statistic.

**Results:**

Of 265 citations, a total of 10 eligible studies containing 30,994 participants were finally included. Analysis of these studies showed that there was a significant association between periodontitis and OSA (OR=2.17, 95% CI: 1.66-2.83), with no significant heterogeneity between the studies (I2=42.7%, *p*=0.073). Analysis of three surveys indicated that periodontitis is significantly associated with mild-to-moderate OSA (OR=2.51, 95% CI: 1.32-4.78; I2=0.0%, *p*=0.527), but not with severe OSA (OR=1.58, 95% CI: 0.70-3.58; I2=0.0%, *p*=0.469).

**Conclusions:**

According to the results, periodontitis has a direct association with OSA. Also, periodontitis has been shown to be associated with mild-to-moderate OSA, but not with severe OSA. Further studies are warranted to elucidate the mechanisms of these associations.

** Key words:**Periodontitis, obstructive sleep apnea, systematic review.

## Introduction

Periodontitis is a chronic infection of the tissue surrounding the tooth mainly caused by bacterial pathogens. The prevalence of periodontitis has been reported as about 11% among the adults worldwide (1). In this condition, the host immune responses lead to secretion of various inflammatory mediators (such as tumor necrosis factor-alpha [TNF-α], interleukin-6 [IL-6], IL-1B, and IL-33), resulting in destruction of the tooth structure, and ultimately tooth loss (2,3). Releasing the mediators into the blood and circulating via the vascular system can also impact other parts of the body, potentially contributing to the development of other diseases such as diabetes, rheumatoid arthritis, coronary heart disease, and recently obstructive sleep apnea (OSA) (4).

OSA is the most common sleep-related breathing disorder (affecting 2-4% of the adults), characterized by periodic and repetitive upper airway collapse leading to oxygen desaturation and arousals (5). It was reported that the levels of inflammatory mediators (such as TNF-α, IL-6, and IL-1B) are higher in cases with OSA (6), and that was why the researchers proposed a plausible association between OSA and other inflammatory conditions, such as periodontitis. In this regard, there are a number of studies reporting that periodontitis is directly associated with OSA (7,8); however, some other surveys did not demonstrate the same results about this subject (9,10). To clarify this issue, it is therefore necessary to conduct a comprehensive literature review on the available published data.

In the present study, we performed a contemporaneous systematic review and meta-analysis of studies investigating the association between periodontitis and OSA. We also tried to find out whether periodontitis is associated with different severities of OSA.

## Material and Methods

-Information sources and search strategy

We searched the literature published from the inception to 31 December 2021 from the databases of Embase, PubMed, Scopus, and Web of Science, using a combination of the keywords “apnea” OR “sleep apnea” AND “periodontal” OR “periodontitis”. The search was limited to Title or Abstract. No language restriction was applied. We also conducted a hand search of the reference lists of the retrieved papers for additional sources.

-Inclusion and exclusion criteria

We included observational studies recruiting adults (≥18 years old) that evaluated the association between periodontitis and OSA. The diagnosis of periodontitis could be confirmed as per the 2017 classification system of periodontal and peri-implant diseases and conditions (11), or the 1999 Consensus Classification System of Periodontal Disease (12) was considered. The diagnosis of OSA could be based on polysomnography or a validated questionnaire. The exclusion criteria were as follows:

1. Reviews, case reports, editorials, and letter to the editors.

2. Duplicate papers.

3. Surveys recruited children.

4. Studies that did not clearly explain the population characteristics (e.g., age), periodontitis definition, study outcomes, etc.

-Study selection and data extraction

Two investigators (NK and MZ) screened independently the titles and abstracts of the identified articles for potential suitability. Full-texts of the potential papers were assessed for the final suitability judgment for inclusion. Any disagreements were resolved by the consensus between the reviewers. Finally, the following data were extracted onto a Microsoft Excel spreadsheet (Microsoft Corporation, Redmond, Washington) by the two authors (NK and MK): first author’s name, publication year, country, total number of subjects, mean age, number of men and women, method used for diagnosis of OSA, number with or without periodontitis, number with or without OSA. The data related to the association between periodontitis and OSA severity were also recorded, if available; in this regard, we extracted the proportion of periodontitis among various categories OSA severity (mild-to-moderate, and severe), or the correlation coefficient between periodontitis and OSA severity. Non-English papers were translated using Google Translate.

-Risk of bias assessment

For the quality assessment of the included studies, we used the Newcastle–Ottawa scale (NOS) for non-randomized studies (13,14). The NOS adapted for cross-sectional studies attributed a score ranging from 0 to 10 to each study (≥6 points = high quality, 3-5 points = moderate quality, ≤2 points = low quality), and the NOS for case-control studies attributed a score ranging from 0 to 9 to each study (≥6 points = high quality, 3-5 points = moderate quality, ≤2 points = low quality).

-Study outcomes and statistical analysis

We compared the prevalence of periodontitis between OSA-positive and negative individuals using a pooled odds ratio (OR) with a 95% confidence interval (CI). A subgroup analysis was also performed according to the method of OSA confirmation. To evaluate the association between periodontitis and OSA severity, two analyses were conducted; first, the prevalence of periodontitis was compared between the OSA-negative individuals and cases with mild-to-moderate and severe OSA, and second, the correlation coefficients between Clinical Attachment Level/Loss (CAL) and Apnea-Hypopnea Index (AHI) were pooled to estimate the magnitude correlation between the severity of the two conditions. Random-effects model was used to pool data to give more conservative estimates. The heterogeneity between the studies was examined by the I2 statistic and the chi-squared test with a *p-value* <0.10 considered as a threshold for a statistically significant degree of heterogeneity (15). The publication bias was explored using a funnel plot and Egger’s test, where there were at least 10 studies identified (16,17). Meta-regression was used to assess the potential impact of publication year on the outcome, with a *p-value* <0.05 considered statistically significant. All statistical analyses were done using the R package “meta” (https://cran.r-project.org/package=meta).

## Results

-Search results, study selection and characteristics

**Figure 1:**
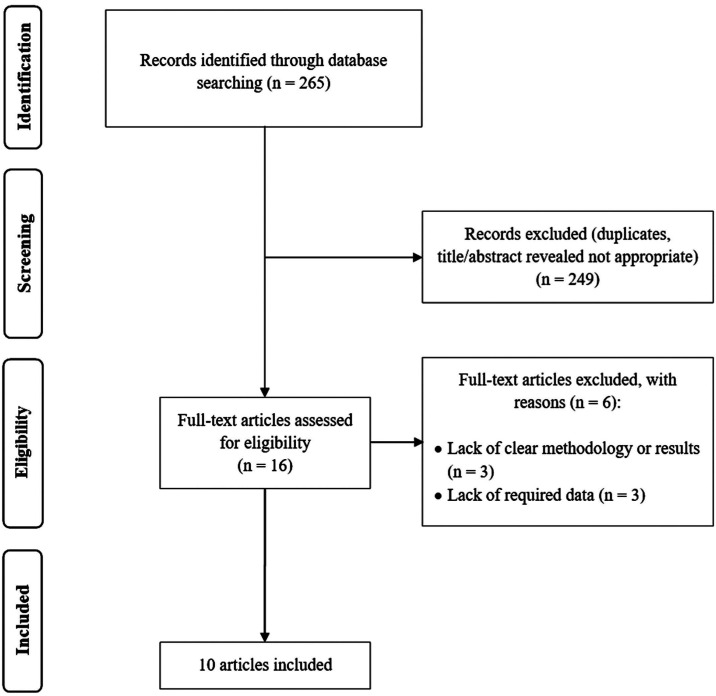
PRISMA flowchart.

The search strategy initially recovered 265 citations. After excluding duplicates, as well as those not meeting the inclusion criteria, a total of 10 studies containing 30,994 participants were finally included (7-10,18-23). The search results have been represented in a flow diagram as per the PRISMA (Preferred Reporting Items for Systematic Review and Meta-Analysis) guideline (Fig. 1) (24). All articles included were written in English. The agreement between the investigators for the eligibility judgment was excellent (Kappa statistic=0.88). Three studies had a high quality (8,10,21) and seven studies had a moderate quality (7,9,18-20,22,23). The baseline characteristics, as well as quality assessment, of the included papers have been summarized in Table 1.

**Table 1:**
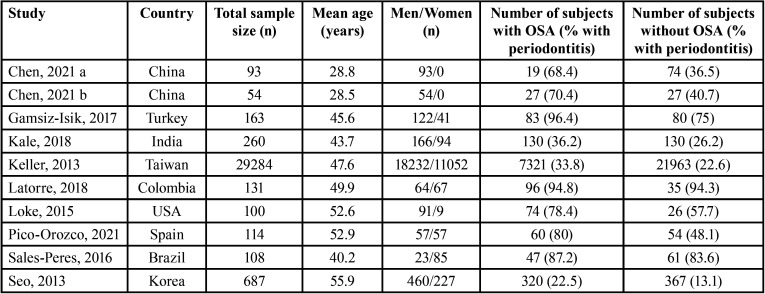
Characteristics of studies reporting prevalence of periodontitis according to obstructive sleep apnea (OSA) status.

-Periodontitis and obstructive sleep apnea

**Figure 2:**
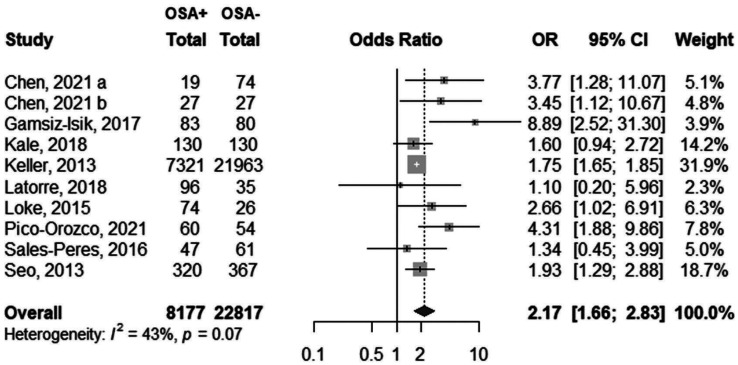
Forest plot of odds of periodontitis according to obstructive sleep apnea (OSA) status.

**Figure 3:**
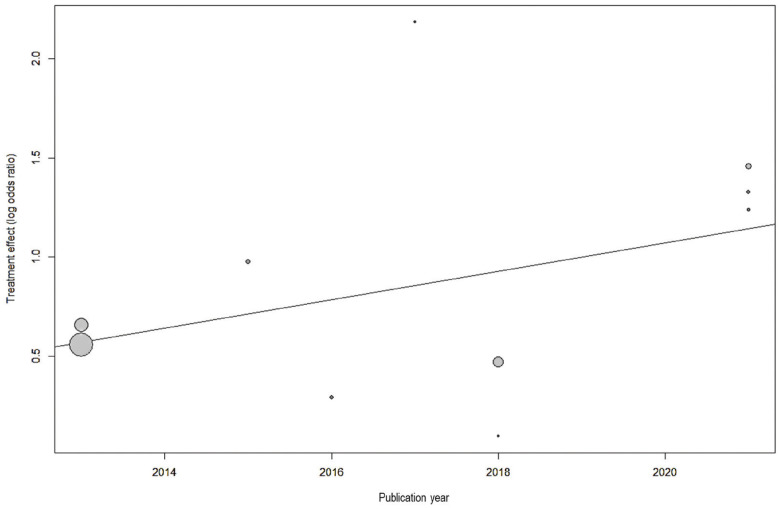
Funnel plot to assess publication bias across the studies evaluating the association between periodontitis and obstructive sleep apnea.

**Figure 4:**
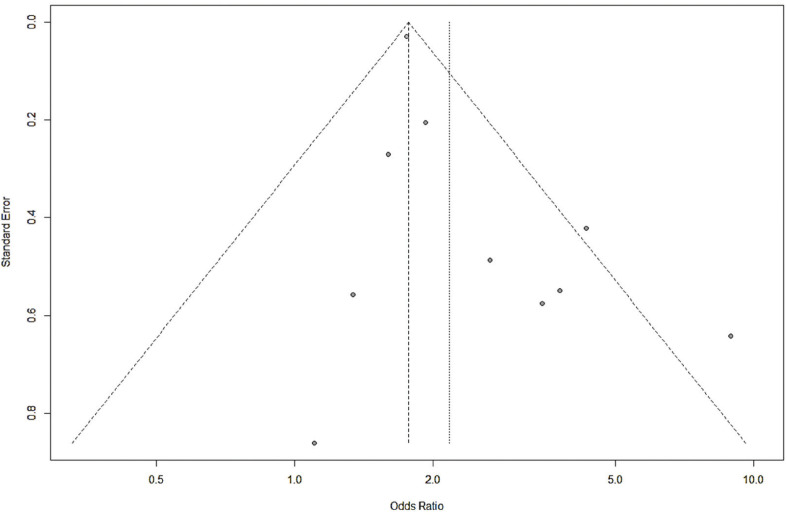
Meta-regression to assess the effect of publication year on the study outcome.

Analysis of 10 studies showed that there was a significant association between periodontitis and OSA (OR=2.17, 95% CI: 1.66-2.83), with no significant heterogeneity between the studies (I2=42.7%, *p*=0.073) (Fig. 2). Visual inspection of funnel plot did not indicate asymmetry (Fig. 3). Egger’s test also did not show significant publication bias (*p*=0.065). Meta-regression analysis demonstrated that publication year (β=0.072, *p*=0.056) did not explain the heterogeneity in the outcome (Fig. 4).

There were three individual studies reporting the estimates adjusted for possible confounders (sex and age) (8,20,21); pooling these data still showed a direct association between periodontitis and OSA (OR=1.75, 95% CI: 1.65-1.85), without significant heterogeneity between the studies (I2=0.0%, *p*=0.610).

For validation of OSA diagnosis, seven studies used polysomnography with a pooled OR of 1.31 (95% CI: 1.20-1.43; I2=12.2%, *p*=0.336) (7,9,18-22), one study used AHI with an OR of 1.59 (95% CI: 1.13-2.23) (8), another study used STOP-Bang Questionnaire with a non-significant OR of 1.28 (95% CI: 0.87-1.89) (23), and another one used a self-reported measure with a non-significant OR of 1.02 (95% CI: 0.76-1.38) (10).

There were three studies investigating the association between periodontitis and OSA severity (7,9,22). Analysis of these surveys indicated that periodontitis is significantly associated with mild-to-moderate OSA (OR=2.51, 95% CI: 1.32-4.78; I2=0.0%, *p*=0.527); on the other hand, no significant association was identified between periodontitis and severe OSA (OR=1.58, 95% CI: 0.70-3.58; I2=0.0%, *p*=0.469). In addition, there were three other studies evaluating the correlation between CAL and AHI (7,18,22); pooling these data demonstrated that periodontitis had a non-significant positive correlation with OSA severity (r=+0.277, *p*=0.062; I2=47.8%, *p*=0.147).

## Discussion

So far, different studies have tried to elucidate whether periodontitis has an association with OSA. In the present systematic review and meta-analysis, we collected those published data to reach a conclusive result on this topic. Analysis of 10 studies indicated that periodontitis is directly associated with OSA (OR=2.17). This positive association remained when the analysis was restricted to the studies reporting the estimates adjusted for sex and age (OR=1.75). Again, a similar result was observed when we performed the analysis only on the studies used polysomnography (not subjective questionnaires) for OSA assessment (OR=1.31). Regarding the association between periodontitis and OSA severity, we found that periodontitis is directly associated with mild-to-moderate OSA, but not with severe OSA; on the other hand, pooling the correlation coefficients showed that periodontitis severity is positively correlated with OSA severity, but the correlation was not statistically significant. Our findings support the results of the review by Al-Jewair *et al*. (25), in which the authors reported that periodontitis has a direct association with OSA (OR=1.66); on the other hand, they did not find a significant association between periodontitis and OSA severity, which was inconsistent with our results.

Previous studies suggested different likely mechanisms to explain the association between periodontitis and OSA. First, intermittent hypoxia observed in the OSA can provide conditions for oxidative stress and systemic inflammation, as the damage of hypoxia to the periodontal tissue was also seen in the in-vitro studies (26,27). Second, drying of the oral cavity as a result of chronic open breathing can prevent its self-cleaning, potentially leading to periodontitis progression (28). Finally, as mentioned earlier, both of periodontitis and OSA are linked to the systemic inflammation, which can partially explain the association between these two conditions. However, more studies need to be done to reach a consensus on the given mechanisms.

We tried to conduct a comprehensive literature search in various databases using several keywords. A recursive search of the references of the eligible papers was also performed to minimize the probability of missing the pertinent articles. Eligibility judgment of the studies was performed by two independent authors. Additionally, risk of bias assessment was done for each study. For all analyses, we used random-effects model for pooling the data for more conservative estimates. Publication bias was also assessed where sufficient studies existed. In addition, we performed meta-regression analysis to investigate the potential influence of publication year on the study outcome. ‘Study date’ could potentially impact the heterogeneity and study outcomes, due to differences in experience and knowledge of the clinicians toward apnea and periodontitis, as well as differences in periodontitis diagnostic criteria during the past years; however, considering that study date was not reported in some studies, we preferred to use ‘publication year’ instead. Finally, subgroup and sensitivity analyses were caried out to decrease the effect of heterogeneity on the results; of course, our findings showed that the heterogeneity between the studies was mostly low or moderate, and no significant heterogeneity was found between the studies.

The present study had some limitations. First, wide 95% CIs were observed for some of the pooled estimates, owing to small sample size of some of the studies. Second, the number of studies reporting the adjusted estimates of the association was limited, and therefore, we had to rely on the raw data from the surveys. Finally, the total number of studies included was not large enough and it is proposed to design and perform new studies to catch a more reliable conclusion on the association between periodontitis and OSA.

In conclusion, the results of this meta-analysis demonstrated that periodontitis has a direct association with OSA. We also found that periodontitis is associated with mild-to-moderate OSA, but not with severe OSA. Further studies are warranted to elucidate the mechanisms of these associations.
